# Supplementary material for: Unraveling gut microbiome alterations and metabolic signatures in hereditary transthyretin amyloidosis
Source: Microbiol Spectr. 2025 May 23;13(7):e02302-24. doi: 10.1128/spectrum.02302-24 (PMC12210864; doi:10.1128/spectrum.02302-24)
Supplement: Supplemental figures — Figures S1 to S3. [file spectrum.02302-24-s0001.pdf]

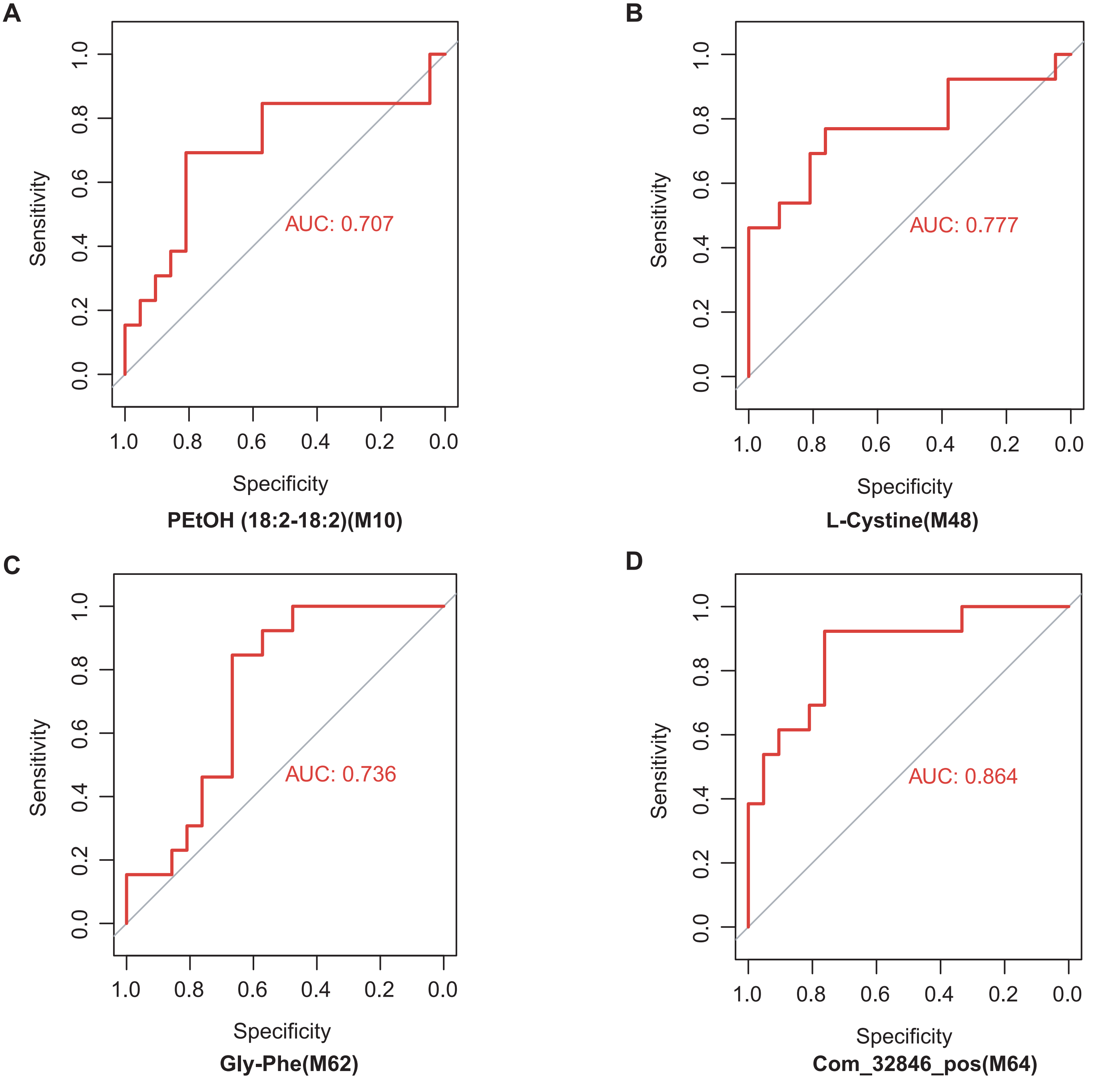

Figure S1. Receiver operating characteristic curve (ROCs) of additional metabolite modules which correlate to cardiac amyloidosis. (A) M10: PEtOH. (B) M48: L-Cystine. (C) M62: Gly-Phe. (D) M64: Com_32846_pos = 1-methyl-3,5-di(1-naphthylmethylidene)piperidin-4-one. Area under ROCs (AUCs) is shown. hATTR versus HC.

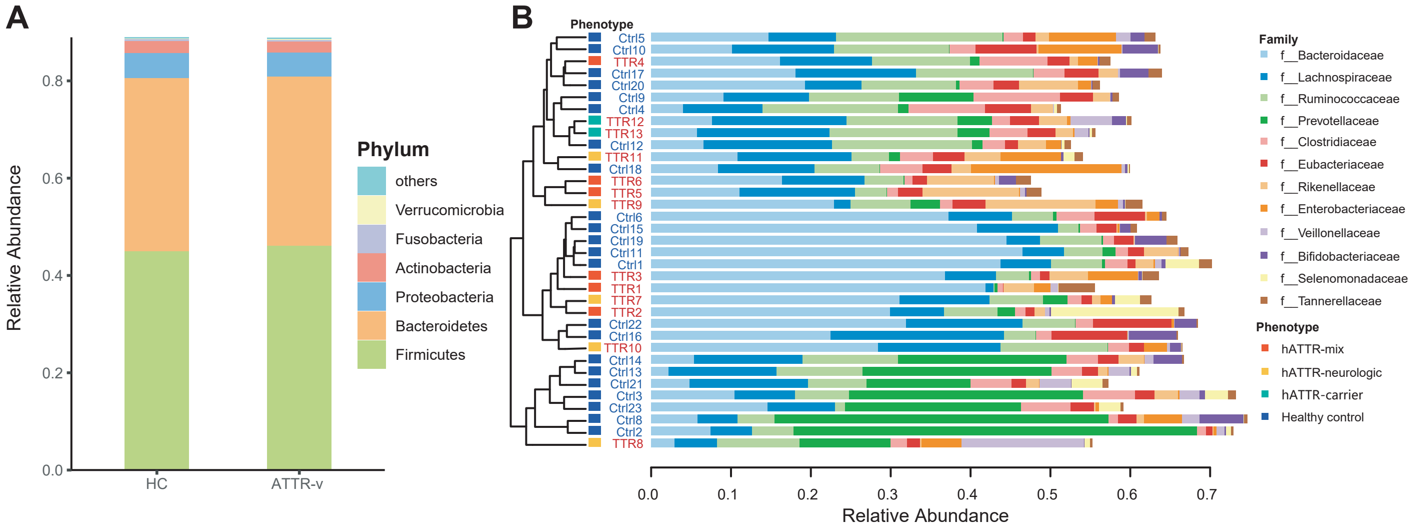

Figure S2. Overview of microbial compositions in different groups. (A) Average relative abundance of phyla in hATTR and HC groups. (B) Distribution of families across samples and hierarchical clustering of samples based on Bray-Curtis distance. Samples from the hATTR group are highlighted in red. Samples from the HC group are highlighted in blue. Phenotypes of hATTR are marked by different color blocks, as shown in the lower right corner.

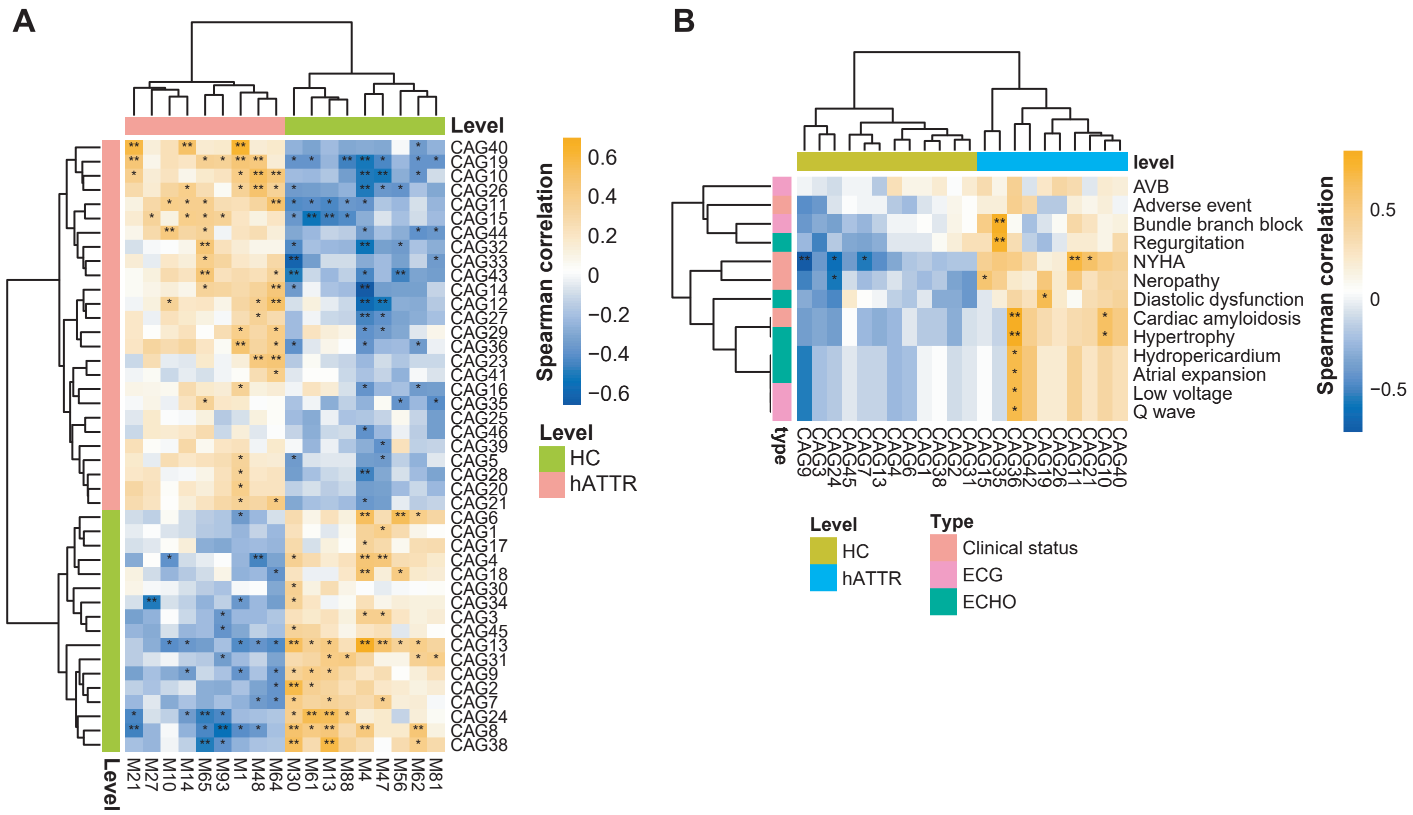

Figure S3. Multi-omics correlation analyses. (A) The heatmap illustrates correlations between CAGs and serum metabolite modules. (B) The heatmap illustrates correlations between CAGs and clinical indices. Spearman's rank correlation. *P-value < 0.05, **P-value < 0.01.
