# Supplementary material for: Unraveling gut microbiome alterations and metabolic signatures in hereditary transthyretin amyloidosis
Source: Microbiol Spectr. 2025 May 23;13(7):e02302-24. doi: 10.1128/spectrum.02302-24 (PMC12210864; doi:10.1128/spectrum.02302-24)
Supplement: Supplemental methods — Additional methods. [file spectrum.02302-24-s0002.docx]

**Additional file 3**

**Supplementary methods**

**Unraveling Gut Microbiome Alterations and Metabolic Signatures in Hereditary Transthyretin Amyloidosis**

**Collection of blood samples**

Peripheral fasting blood was drawn in the morning. Peripheral blood samples were centrifuged at 3000 rpm for 5 min after standing at room temperature for at least 30 min, and the supernatant was purified. Serum samples were then stored at − 80 °C immediately.

**Collection of stool samples**

Participants were given a stool sampler and provided detailed illustrated instructions for sample collection. Stool samples freshly collected from each participant were immediately transported to the laboratory and frozen at − 80 °C immediately.

**DNA extraction by CTAB method**

1. Pipette 1000 µL of CTAB lysis buffer into a 2.0 mL EP tube, add lysozyme, and then add an appropriate amount of sample to the lysis buffer. Incubate in a water bath at 65°C, inverting the tube several times during the process to ensure complete lysis of the sample.
2. Centrifuge and collect the supernatant. Add phenol (pH 8.0): chloroform: isoamyl alcohol (25:24:1), invert to mix thoroughly, and centrifuge at 12,000 rpm for 10 minutes.
3. Collect the supernatant again. Add chloroform: isoamyl alcohol (24:1), invert to mix thoroughly, and centrifuge at 12,000 rpm for 10 minutes.
4. Transfer the supernatant to a 1.5 mL centrifuge tube, add isopropanol, and mix by inverting. Precipitate at -20°C.
5. Centrifuge at 12,000 rpm for 10 minutes. Carefully remove the liquid without disturbing the precipitate. Wash the precipitate twice with 1 mL of 75% ethanol. Any remaining liquid can be removed by centrifugation again and then aspirated with a pipette tip.
6. Dry the precipitate in a laminar flow hood or at room temperature (do not over-dry the DNA sample, as it may be difficult to dissolve).
7. Dissolve the DNA sample in ddH₂O. If necessary, incubate at 55-60°C for 10 minutes to aid dissolution.
8. Add 1 µL of RNase A to digest RNA, and incubate at 37°C for 15 minutes.

**Library preparation**

We analyzed the integrity and purity of DNA using agarose gel electrophoresis (AGE) and precisely quantified the DNA concentration using a Qubit fluorometer. For qualified DNA samples, we randomly fragmented them into approximately 350 bp fragments using a Covaris ultrasonicator. Subsequently, the library preparation was completed through a series of steps, including end repair, A-tailing, adapter ligation, purification, and PCR amplification. After the DNA library construction was completed, we performed an initial quantification using Qubit 2.0, diluted the library to 2 ng/µL, and then assessed the insert size of the library using an Agilent 2100 Bioanalyzer. Once the insert size met the expected range, we used quantitative PCR (qPCR) to accurately determine the effective concentration of the library, which must be greater than 3 nM to ensure library quality.
